# Application of the Semi-Automatic Titration Method Using a Webcam for the Determination of Calcium in Milk and Dairy Products

**DOI:** 10.3390/molecules30173553

**Published:** 2025-08-30

**Authors:** Alexander Shyichuk, Dorota Ziółkowska, Iryna Shyychuk, Maria Kowalska

**Affiliations:** 1 Faculty of Chemical Technology and Engineering, Bydgoszcz University of Science and Technology, Seminaryjna 3, 85-326 Bydgoszcz, Poland; szyjczuk@pbs.edu.pl (A.S.); iryna.shyychuk@pbs.edu.pl (I.S.); maria.kowalska@pbs.edu.pl (M.K.); 2 Educational and Scientific Center of Materials Science and Nanotechnology, Vasyl Stefanyk Precarpathian National University, 76018 Ivano-Frankivsk, Ukraine

**Keywords:** food analysis, complexometry, EDTA titration, RGB image, calcein, hydroxy naphthol blue

## Abstract

Quantitative determination of calcium content in milk and dairy products is an important analytical task due to the great importance of this element in the human diet. Calcium determination in milk and dairy products was performed using semi-automatic complexometric titration with a webcam as a color change detector. The color changes were registered directly in turbid dairy dispersions, creating a white background. The analytical signals tested were the color component (R, G, B) read from the titration beaker images and the calculated Hue parameter. For the calcein indicator, the optimal signals are Green and Hue. For the HNB indicator, the optimal signals are Red and Hue. The developed method of titration using an RGB camera detector is characterized by excellent linearity (R^2^ = 0.9999) and accuracy. RSD values range from 0.3 to 2.9%. Recovery values range from 105 to 113%. Examples of calcium determination in commercial products include milk, fermented products, cream, and cottage cheese.

## 1. Introduction

Calcium is an essential element for the human body, especially for the skeletal, muscular, and circulatory systems [1,2,3]. The need for calcium depends on age, physical activity, disease states, etc. [4]. If the body is not supplied with the right amount of calcium, it draws its reserves from the bones, resulting in increased bone fractures [5,6]. In a typical diet, the main source of calcium is dairy products, but fish and plant foods are also important [7,8]. The calcium content in milk is quite high (usually 120 mg in 100 mL of cow milk) [9], while yogurts can be even richer in calcium (usually 100–180 mg/100 g) [2,10,11]. The source of the increased calcium content in yogurts compared to raw milk is the milk powder added during production [11]. On the other hand, excessive calcium intake can cause kidney strain, excessive thirst, constipation, and fatigue [12]. Therefore, monitoring the calcium content in food products is an important analytical task.

There are many analytical methods for the quantitative determination of calcium in dairy products, and each has its advantages and disadvantages. Currently used methods for determining calcium in dairy products include atomic absorption spectroscopy (AAS), inductively coupled plasma spectroscopy (ICP-OES and ICP-MS), ion-selective electrodes (ISEs), and complexometric titration. In addition, ion exchange chromatography (IEC, IEX), laser-induced breakdown spectroscopy (LIBS), infrared spectroscopy (NIR, MIR), and X-ray fluorescence spectroscopy (XRF, EDX) are used [13,14].

Among the spectroscopic methods, AAS is commonly used [13]. The AAS method enables the determination of many dairy macroelements (Ca, Mg, Na, K, P), although this is not performed simultaneously. AAS is less expensive and less complex than other spectral techniques, but it also has limitations. Particularly troublesome is the multi-stage sample preparation (usually involving ashing and then dissolving in mineral acid), which significantly extends the analysis time. A more advanced technique is ICP [13]. At very high temperatures, the sample is ionized, and the excited atoms and ions emit light with a characteristic wavelength, the intensity of which depends on the concentration of the element. Unlike AAS, it enables simultaneous, precise determination of a larger number of elements, even in trace amounts. In laser-induced breakdown spectroscopy (LIBS), the sample is excited by a high-energy pulsed laser beam, and the emitted light is measured spectrophotometrically [14]. No sample preparation is required, and a short measurement time makes LIBS suitable for real-time control of dairy products in industrial technologies.

X-ray methods (XRF, EDX) are not as sensitive as ICP, but they allow for the determination in a relatively short time (up to 60 min) without the use of acids or organic solvents. However, due to the high cost of the equipment, they are less frequently used for analyzing dairy products than AAS and ICP [13]. In instrumental neutron activation analysis (INAA), the sample is activated by irradiation with reactive neutrons, and the induced radioactivity is measured using a gamma-ray spectrometer. Despite the lack of sample pre-treatment, this method has limited industrial application due to the high cost of the equipment and the high qualifications required from the operator [14].

In contrast, infrared (IR) spectroscopy in the near (NIR) and mid (MIR) infrared ranges is widely used in the dairy industry for both quantitative and qualitative analysis. Generally, analytical infrared techniques are characterized by high predictive power thanks to their coupling with statistical (chemometric) programs for spectral interpretation. Literature reports indicate that the IR technique for analyzing milk and dairy products requires improvement [14]. When determining the content of macro- and micro-minerals in milk, IR spectroscopy can be supported with machine learning algorithms [15]. The results achieved in predicting milk composition were described as moderate or good.

Unlike spectral methods, which allow for the determination of total calcium, ISEs and IEC measure calcium in ionic form. The determination is based on the interaction of ionic calcium with an ion exchange resin. The ISE method requires the preparation of calibration solutions with similar physicochemical properties to the sample (e.g., ionic strength, concentration, and pH), but the sample itself does not require preparation. The determination procedure is cheap in terms of labor input. Unlike ISEs, IEC enables the simultaneous, accurate determination of different ions, but it is more expensive and time-consuming. Therefore, it is used sporadically [14]. Many analytical methods considered simple, reliable, and inexpensive, e.g., gravimetry, capillary electrophoresis, coulometry, voltammetry, UV-VIS spectrophotometry, or spectrofluorimetry [16], are not suitable for analyzing milk and dairy products.

The method of complexometric titration is reliable, simple to use, and does not require any expensive equipment. However, determining calcium in dairy matrices, especially cheeses, can be a challenge. The first problem is interference from magnesium [17]. As the standard EDTA (ethylenediaminetetraacetic acid) titrant is a strong and non-selective complexone, the analyte solution must be made alkaline to precipitate magnesium. Alternatively, ethylene glycol-bis(2-aminoethyl)-tetraacetic acid (EGTA) can be used, which is more specific to calcium [13]. Another problem is the high turbidity of the analyzed solution, which makes it difficult to visually recognize the endpoint of the titration [17]. The blurred color change at the titration endpoint can lead to a subjective error in the amount of titrant used [13]. Typical indicators for the complexometric titration include the purpuric acid salt (murexide), eriochrome blue S E (solochrome), and 2-hydroxy-l-(2-hydroxy-4-sulfo-lnaphthylazo)-3-naphthoic acid [17,18].

To completely release calcium from the sample and eliminate sample turbidity, the classic titration method requires precipitation of the protein with trichloroacetic acid or salicylic acid. In the first case, calcium is precipitated as oxalate and titrated with potassium permanganate. In the second case, calcium is titrated directly with EDTA in the presence of the palladiazo indicator [14]. Another technique for preparing cheese samples involves ashing and dissolving in dilute acid. Calcium chloride is then added, and back titration is performed with EDTA using hydroxy naphthol blue as an indicator [17]. Due to the need for preliminary preparation of dairy samples, the classic titration methods are time-consuming and labor-intensive [14].

The main advantages and disadvantages of different techniques used for calcium determination in milk and dairy products are summarized in Table 1.

The complexometric titration method can be used to determine calcium in dairy products (milk, cheese, yogurt) with potentiometric detection of the endpoint using a Cu ion-selective electrode [19]. The titrant is EGTA, and the indicator is the copper complex of the titrant. The advantage of this method is that it does not require pre-treatment of milk and yogurt samples. However, the consumption of the analyte sample is large (10 g of milk, yogurt, etc., or 1 g of cheese) [19].

Detection of indicator color change at the titration endpoint can be improved using a digital camera that perfectly recognizes subtle color differences. RGB images were reported to be useful for detecting color changes at the endpoint in the complexometric determination of water hardness with the Eriochrome Black T indicator [20,21].

Recently, camera image analysis has been used to detect the endpoint of titration using the calcein indicator [22]. The calcein indicator was used in two measurement modes: fluorescence and reflectance. The webcam proved to be a cheap and convenient signal detector in colorimetric titration. Analysis of the recorded digital images allowed for the selection of optimal color components appropriate for the indicator color change and imaging conditions. The method has been proven to be linear over a wide range of calcium concentrations (0.05–2.5 mM). RSD values are 0.7–3.1% for high calcium concentrations and 3.5–6.6% for low calcium concentrations. Recovery values range from 103 to 108%. The Limit of Detection and the Limit of Quantitation are 2 μmol/L and 6 μmol/L, respectively. The method was tested by determining calcium in commercial mineral waters with different mineral content. The semi-automatic titration procedure using EDTA and calcein proved to be environmentally friendly [22].

In this work, a semi-automatic titration set with a webcam detector was adapted for the quantification of calcium in dairy products. Fortunately, turbid dairy dispersions create a white background, making it easier to detect color changes with a webcam. Distinctive endpoints were recorded using calcein and hydroxy naphthol blue indicators. The usefulness of the method was checked by determining calcium in various types of milk and commercial dairy products.

## 2. Results and Discussion

### 2.1. Detecting the Endpoint Using Calcein as an Indicator

**Figure 1:**
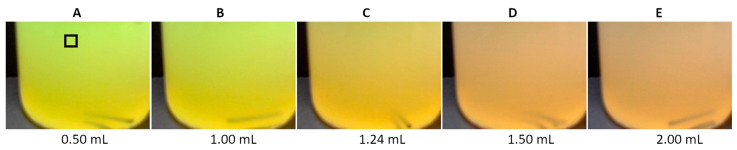
Images of the titration beaker recorded during the titration of an aqueous dispersion containing 1 mL of milk, using calcein of 8 μmol/L as an indicator. Volumes of EDTA are given. The black square in photo (**A**) indicates the color sampling area.

It has been previously demonstrated [22] that calcein is a suitable indicator for determining calcium in water by the complexometric method, utilizing a camera as a signal detector. It was found that calcein is also suited for determining calcium in aqueous milk dispersions. Figure 1 illustrates color changes during the titration of a 1% *v*/*v* milk dispersion in water with EDTA using calcein indicator. Initially, the fluorescent calcein–Ca complex produces a yellow-green (R/G/B of 206/223/89) color under LED light (Figure 1A). After all calcium ions are complexed with EDTA, the color changes to dirty orange (R/G/B of 205/168/114) (Figure 1E).

The color of calcein in milk–water dispersions is yellow-green (R/G/B between 206/223/89 and 206/216/88) with moderate fluorescence (Figure 1A,B). For comparison, the color of calcein in water is lime green (R/G/B of 90/254/93 at moderate calcein concentration), with strong fluorescence [22]. The transition and final colors (Figure 1C–E) resemble those characteristic of titration in reflectance mode rather than fluorescence mode [22]. This is probably due to the turbid medium partially quenching fluorescence. For this reason, the analyte solution should be highly diluted with deionized water, and the color should be sampled at the top of the image (Figure 1A). As previously shown [22], the size of the color sampling area has no significant effect on the reading of EDTA volume at the titration endpoint. To obtain titration graphs with less noise, it is recommended to select a larger area at the top of the beaker image.

**Figure 2:**
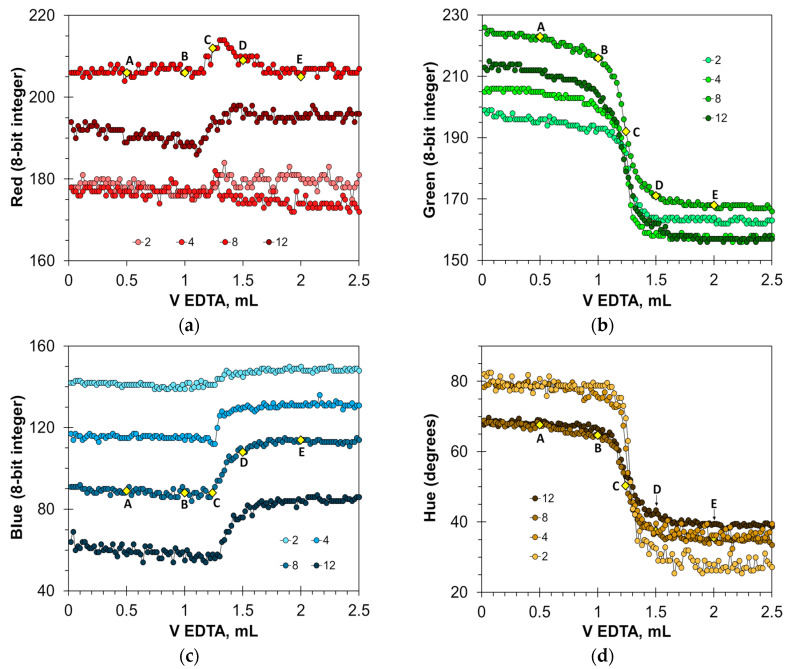
Titration graphs of a water solution containing 1 mL of milk obtained using calcein as an indicator. The Red (**a**), Green (**b**), Blue (**c**), and Hue (**d**) values are plotted vs. EDTA volume. The indicator concentrations are shown in μmol/L. Notations A–E correspond to those in Figure 1.

Due to the sharp color change in calcein, the Green and Hue signals are well-suited to detect the titration endpoint (Figure 2b,d). The optimal concentration of the calcein indicator in milk–water dispersion for these signals is from 2 to 12 μmol/L. The magnitude of signal change decreases at calcein concentrations higher than 8 μmol/L (Figure 2b) or 4 μmol/L (Figure 2d). Therefore, the use of higher calcein concentrations is not recommended. The basic Blue color (Figure 2c) changes the values by 30 units, but only with the appropriate selection of calcein concentration, i.e., from 8 to 12 μmol/L. The Red signal is not suitable due to large signal oscillations (Figure 2a). For comparison, in aqueous solutions, the best parameters for endpoint detection were the primary Green and Blue colors at calcein concentrations ranging from 1 to 12 μmol/L [22].

**Figure 3:**
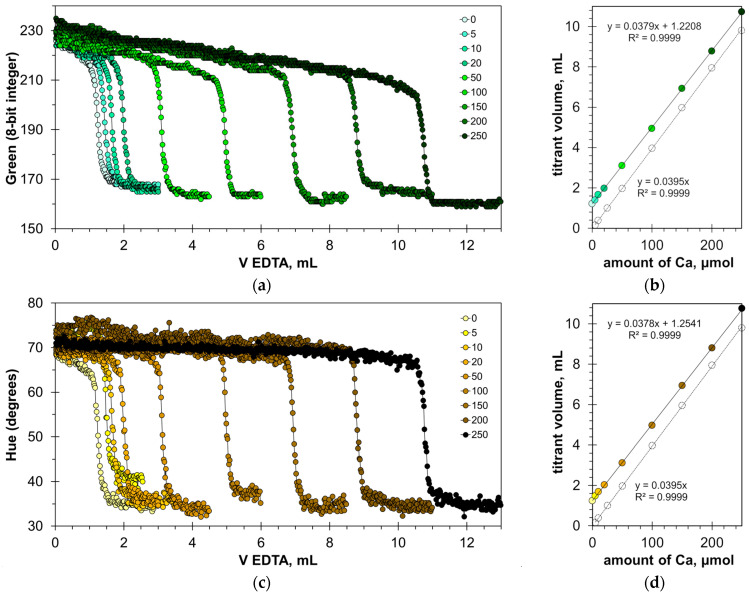
Graphs of the Green (**a**) and Hue (**c**) values vs. EDTA volume obtained in the water solution containing 1% *v*/*v* of milk with the addition of a calcium ion, using the calcein indicator of 8 μmol/L. The amounts of calcium ions are indicated in μmol. The dependences of titrant volume on calcium amount derived for Green (**b**) and Hue (**d**) signals; colorless points correspond to water solutions of calcium.

The found optimal calcein concentrations allow for a reliable detection of the titration endpoint. Figure 3a,c and Figure S1a show the titration graphs obtained for milk dispersion of 1% *v*/*v* with calcium added in amounts ranging from 5 to 250 μmol. The graphs obtained using Green and Hue signals are quite uniform (Figure 3a,c), while the titration graphs obtained using the Blue signal have significant oscillations (Figure S1a). Despite this, the signal jump of the Blue signal at the titration endpoint is sufficiently sharp to read the endpoint volume. Similarly to the titration of water samples [22], the drop in the Green signal at the endpoint slightly decreases with an increase in calcium concentration (Figure 3a). This suggests that the upper limit of calcium determination is higher than the tested value of 250 μmol.

The graphs in Figure 3b,d and Figure S1b have high coefficients of determination, R^2^ = 0.9999, confirming that the EDTA titration method has excellent linearity. The slopes for the Green, Blue, and Hue signals are similar (0.0379, 0.0380, and 0.0378 mL/μmol, respectively). The slopes differ slightly from those obtained for aqueous calcium solutions [22], probably due to the influence of the milk matrix. The values of graph shift are almost the same for the Green and Hue signals (1.22 and 1.25 mL, respectively) but differ slightly for the Blue signal (1.35 mL). This suggests that the Blue signal slightly overestimates the calcium concentration compared to the Green and Hue signals. This is also evidenced by the position of point C in Figure 2b–d.

### 2.2. Detecting the Endpoint Using Hydroxy Naphthol Blue as an Indicator

**Figure 4:**
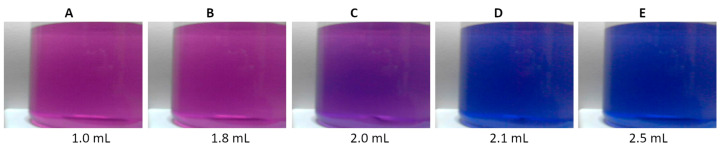
Images of the titration beaker recorded during the titration of a water solution containing 50 μmole of Ca, using hydroxy naphthol blue of 4 mmol/L as an indicator. Volumes of EDTA are given.

The hydroxy naphthol blue (HNB) indicator is a derivative of azonaphtholsulfonic acid. The suitability of the HNB indicator was tested in the reflectance mode. Figure 4 shows photos of the reaction beaker recorded during the titration of an aqueous calcium solution. The indicator changes color from pink (Figure 4A,B, R/G/B of 117/33/114 and 116/33/117, respectively) to blue (Figure 4D,E, R/G/B of 18/31/129 and 13/31/130, respectively). The color at the transition point is purple (Figure 4C, R/G/B of 76/29/118).

**Figure 5:**
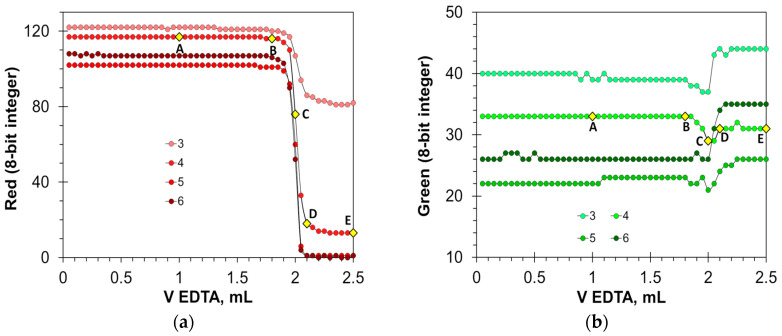

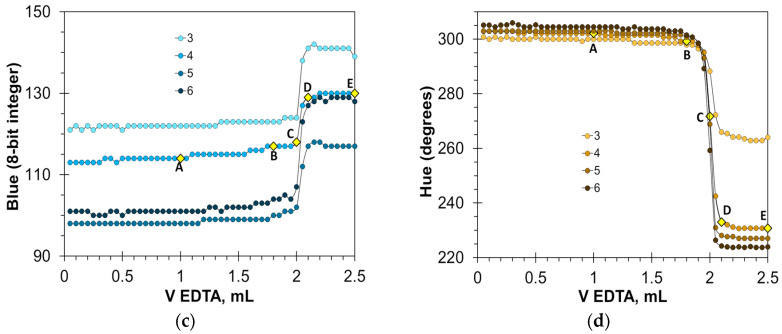
Titration graphs of calcium aqueous solution obtained using hydroxy naphthol blue as an indicator. The Red (**a**), Green (**b**), Blue (**c**), and Hue (**d**) values are plotted vs. EDTA volume. The indicator concentrations are shown in mmol/L. The amount of calcium ions is 50 μmol. Notations A–E correspond to those in Figure 4.

Figure 5 shows that the HNB indicator provides a very sharp color transition. At the endpoint, the Red values fall by 40 to 100 units (Figure 5a), and the Hue values fall steeply by 80 to 100 degrees (Figure 5d). The Blue signal is also suitable for detecting the endpoint (Figure 5c), but in a smaller range of HNB concentrations. The position of point C on the titration curves is the same for the Red, Blue, and Hue signals, resulting in the same value of the titration endpoint. In turn, the Green signal is not suitable for an analytical purpose (Figure 5b). The optimal concentration of the HNB indicator is in the range from 3 to 6 mmol/L. An insufficient amount of the indicator results in reduced signal drop. An excessive amount of the indicator also reduces the signal drop because of the dark shades of the solution.

In full agreement with the titration graph shapes, the HNB provides precise results of calcium determination (Table 2). The Hue parameter enables better precision than the primary Red and Blue colors (Table 2).

**Figure 6:**
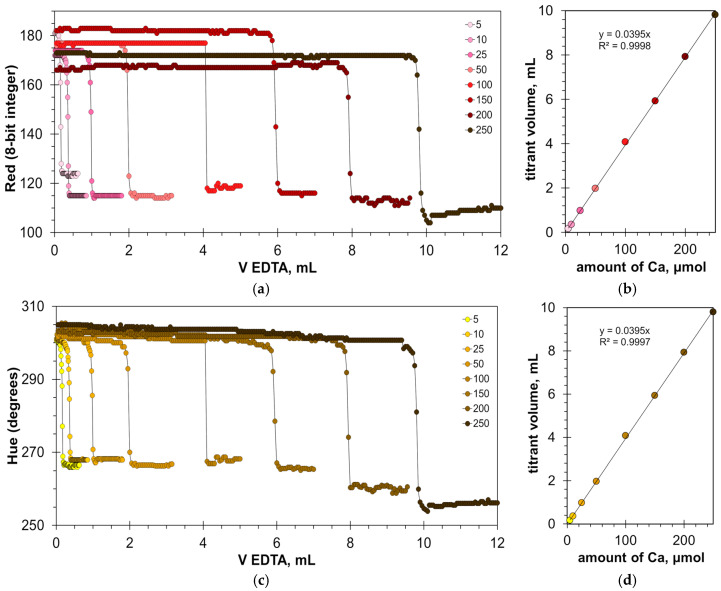
Graphs of the Red (**a**) and Hue (**c**) values vs. EDTA volume obtained using the hydroxy naphthol blue indicator of 4 mmol/L. The amounts of calcium ions in water solutions are indicated in μmol. The dependence of titrant volume on calcium amount derived for Red (**b**) and Hue (**d**) signals.

Another advantage of the Hue parameter is its low dependence on lighting variations. Figure 6 shows a series of titration graphs obtained during the day with varying sunshine and cloud cover. The observed differences in height of the plots of the Red component (Figure 6a) are caused by the changes in light intensity. Advantageously, the Hue graphs have a more uniform character (Figure 6c). Nevertheless, the linearity of the method using Red and Hue signals is the same (Figure 6b,d). Due to its better resistance to varying lighting conditions, the Hue parameter is the most appropriate for automatic endpoint recognition when the HNB indicator is used. Significantly worse results were obtained using the Blue parameter (Figure S2). The Blue parameter showed the greatest discrepancy in results for low calcium concentrations (Table 2). This is due to the small range of variation of the Blue parameter in dilute solutions (Figure S2).

**Figure 7:**
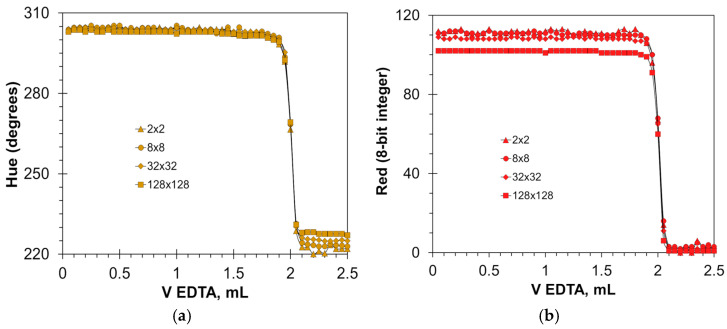
Effect of sampling area dimensions (indicated in pixels) on the titration graphs for Hue (**a**) and Red (**b**) signals. The concentration of the hydroxy naphthol blue indicator is 5 mmol/L. The amount of calcium ions is 50 μmol.

The software used allows for free selection of the color sampling area (Section 3.2). The dimensions of the sampling area have a negligible effect on the sharpness of the color transition (Figure 7). As the color sampling area increases, the titration graphs remain in the same shape, with only the Red signal drop decreasing slightly (Figure 7b). With the reduction in the color sampling area, a slight increase in fluctuations on the flat parts of the Red signal graphs is observed (Figure 7b). In the case of small sampling areas, light reflections and floating bubbles can interfere with the color signals. For this reason, the sampling area for the Red signal should not be smaller than 8 × 8 pixels.

**Figure 8:**
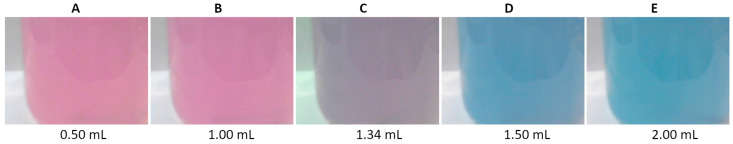
Images of the titration beaker recorded during the titration of 100 mL aqueous dispersion containing 1 mL of milk, using hydroxyl naphthol blue of 4 mmol/L as an indicator. Volumes of EDTA are given.

The HNB indicator is well-suited for calcium determination in dairy products. Figure 8 shows images of the reaction vessel recorded during the titration of an aqueous milk dispersion. The colors of the HNB in the milk matrix are much more pastel than in pure water. In other words, colors are closer to white, as evidenced by higher values of the R, G, and B parameters.

**Figure 9:**
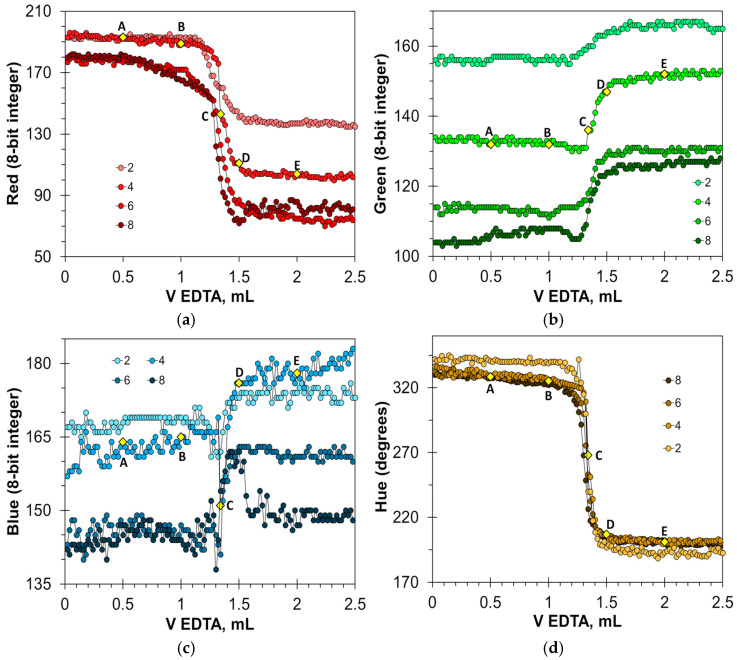
Titration graphs of a water solution containing 1 mL of milk obtained using hydroxyl naphthol blue as an indicator. The Red (**a**), Green (**b**), Blue (**c**), and Hue (**d**) values are plotted vs. EDTA volume. The indicator concentrations are shown in mmol/L. Notations A–E correspond to those in Figure 8.

The indicator changes color from light pink (Figure 8A,B, R/G/B of 193/132/164 and 189/132/165, respectively) to light blue (Figure 8D,E, R/G/B of 111/147/176 and 104/152/178, respectively). At the endpoint of the titration, the color is rather gray-purple (Figure 8C, R/G/B of 143/136/154). This color saturation in a cloudy solution is optimal for the camera, as confirmed by the titration graphs shown in Figure 9.

The changes in the color signals in the aqueous milk dispersion (Figure 9) are less spectacular compared to those in clear aqueous solutions (Figure 5). However, the determination of the endpoint of the titration is reliable. The Red and Hue signals once again proved particularly useful. The Red signal changes by 60–110 units, and the Hue signal by about 140 degrees. The Green signal varies over a smaller range of values (from 10 to 15 for optimal indicator doses), and the change in value at the endpoint is less sharp than in the case of the Red and Hue signals. The location of point C on the titration curve (Figure 9b) suggests that the Green signal may overestimate the assay result. The Blue signal requires precise dosing of the indicator. Only a concentration of 6 mmol/L allows for an accurate reading of the titration endpoint (Figure 9c). It can be concluded that in milk–water dispersions, the HNB indicator is applicable for the endpoint detection using the Red and Hue signals. The optimal concentration range is from 4 to 8 mmol/L.

**Figure 10:**
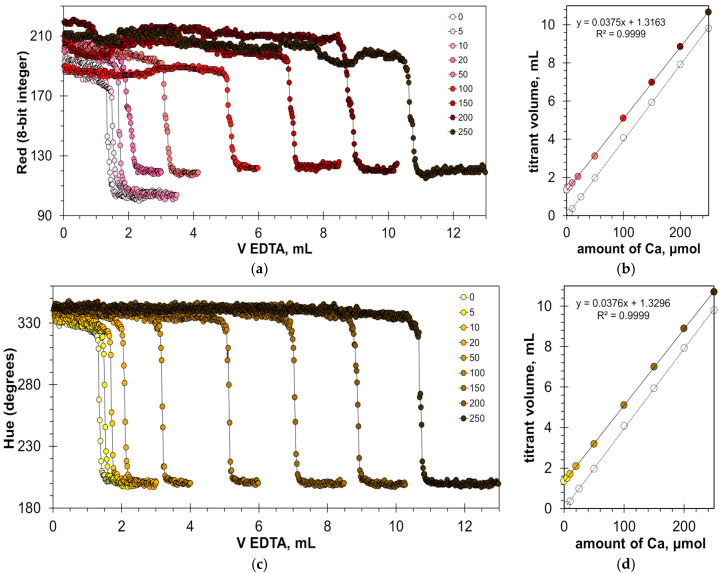
Graphs of the Red (**a**) and Hue (**c**) values vs. EDTA volume obtained in water solution containing 1% *v*/*v* of milk with the addition of a calcium ion, using the HNB indicator of 8 mmol/L. The amounts of calcium ions are indicated in μmol. The dependences of titrant volume on calcium amount derived for Red (**b**) and Hue (**d**) signals; colorless points correspond to water solutions of calcium.

Figure 10a,c show titration graphs obtained for milk–water dispersions with the addition of calcium ions in the range from 5 to 250 μmol. Similar to the titration of calcium in clear water with the HNB indicator (Figure 6a), the range of the Red signal values (Figure 10a) differs for the individual titration curves. This indicates the sensitivity of the measuring system to changes in external illumination but does not affect the position of the titration endpoint (Figure 10b). A similar result was obtained for the Green signal (Figure S3). In the case of the Hue signal, the titration graphs are more uniform (Figure 10c).

Figure 10b,d confirm that the EDTA titration has excellent linearity. The slopes are almost the same and equal to 0.0375 and 0.0376 mL/μmol for Red and Hue signals, respectively. The values of the graph shift are also very close (1.3163 mL and 1.3296 mL for the Red and Hue signals, respectively). This means that the results obtained from these signals will be consistent.

### 2.3. Applicability of the EDTA Titration Method to Dairy Products

Table 3a shows the repeatability of calcium quantification in dairy products, indicating the differences between the four color parameters and the two indicators used. When using the calcein indicator, the suitable color parameters are Hue and Green. When using the hydroxy naphthol blue indicator, the suitable color parameters are Hue and Red.

When using the calcein indicator with the optimal Green and Hue parameters, the RSD values are quite acceptable (1.32% for milk; 1.43% and 2.22% for cream samples). The values of calcium content determined using the Blue signal appear to be slightly overestimated (Table 3a).

The HNB indicator generally provides similar results of calcium determination (Table 3a) for both selected signals: Red and Hue (Figure 9). The results obtained using HNB are slightly higher than those obtained using calcein, but still close to the values indicated by the reference AAS method. The only result obtained using the Green signal seems to be overestimated (Figure 9). The precision of the measurements is similar to, and sometimes slightly better than, the series of measurements using calcein.

The results of complexometric titration using the calcein indicator are consistent with those obtained by the reference AAS method (Table 3a). The results of calcium determination obtained by the titrimetric method appear to be slightly overestimated, whereas the results obtained by the AAS method may be underestimated. This is evidenced by the previously presented results of calcium determination in water solution using calcein (RSD for titration > 100%, and RSD for AAS < 100%) [22] and the results of calcium determination in water solution in the presence of HNB shown in this paper (Table 2, RSD > 100%). Taking this fact into account, it can be assumed that the accuracy of the titration method for determining calcium in dairy products is satisfactory, especially in the following combinations: calcein + Green or Hue signal and HNB + Red signal. The accuracy of the titration method can likely be improved using a wider sample set and eliminating outliers.

Table 3b shows the values of the P parameter obtained from the *t*-test. The *p*-values above 0.05 correspond to the combinations of indicator and color signal that provide statistically significant similarity of the titrimetric results. The *t*-test can also confirm the conclusions from Table 3a about the accuracy of the titration method relative to the reference AAS method. In the case of milk, the discrepancy between the titration results and the AAS measurement is statistically significant. In the cases of cream and yogurt, the titration method and the AAS method give consistent results when using the HNB indicator with the Red analytical signal. In the case of yogurt, comparable results can also be obtained using the calceine indicator and the Green or Hue analytical signal.

It can be concluded that recognizing the titration endpoint with a webcam instead of visual recognition minimizes requirements for operator qualifications and provides accurate results. A similar method of automatic endpoint recognition using a camera uses machine learning on a large set of reaction mixture images [23]. The machine learning approach requires stable lighting conditions to be provided.

### 2.4. The Environmental Friendliness of the Complexometric Semi-Automatic Titration Procedure

**Figure 11:**
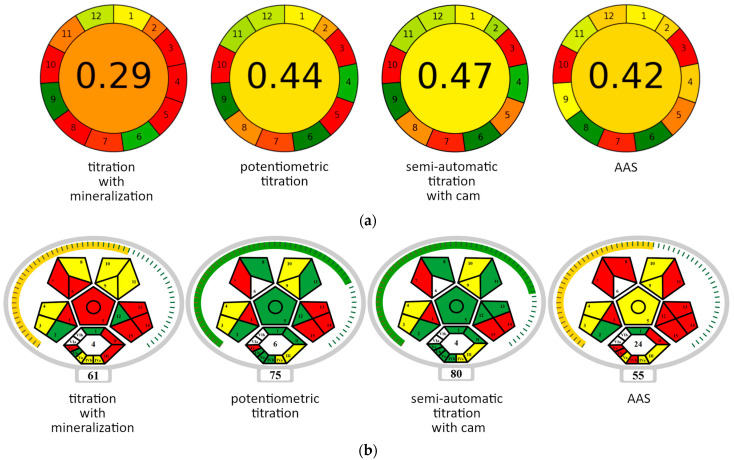
Greenness of the analytical methods assessed according to the AGREE (**a**) and ComplexMoGAPI (**b**) tests. Explanations of the field numbers in the diagrams are provided in Tables S1 and S2 for the AGREE and ComplexMoGAPI tests, respectively. The resultant rating is located in the center of the circle (AGREE test) or below the diagram (ComplexMoGAPI test). The greenness of individual criteria and resultant rating in the AGREE method is marked with smoothly changing colors (from green—the best result to red—the worst result), and in the ComplexMoGAPI method with three colors (green—good result, yellow—moderate result, red—bad result).

The complexometric semi-automatic titration procedure using a camera was evaluated for environmental friendliness with the AGREE [24] and ComplexMoGAPI [25,26] tests. The tests assess analytical methods according to the criteria of Green Analytical Chemistry. Tables S1 and S2a,b list the criteria used and the features assigned to them. Figure 11 presents a summary of the greenness of titration methods and the reference AAS method.

The classic method for determining calcium in dairy products according to the ISO-12081-2010 standard [27] is complexometric titration with prior mineralization. The need to prepare the sample makes this method time-consuming. As a result, the GAPI evaluation assigns low values for four criteria, indicated in Table S1, i.e., 4 (number of steps), 6 (derivatization), 8 (speed of analysis), and 11 (amount of toxic reagents). These criteria are marked in red in Figure 11a. The low scores for other parameters are related to the measurement method itself (titration), which is a manual procedure (criterion 5) and, therefore, off-line (criteria 1 and 3), requires the use of a relatively large amount of analyte (criteria 2 and 5) and generates large amounts of waste (criterion 7). The advantages of this method (marked in green) include moderate toxicity of the reagents (criteria 6 and 12) and low energy consumption (criterion 9).

The method of potentiometric titration has better scores of greenness due to the elimination of the extraction step (Figure 11a, Table S1). By eliminating the analyte preparation step, this method becomes much less labor-intensive and time-consuming (criteria 4 and 8). The consumption of toxic reagents is also drastically reduced (criteria 6 and 11). This results in a 15-point increase in rating. Comparing these methods using the more detailed ComplexMoGAPI test yields a similar ratio of scores (Figure 11b). In the ComplexMoGAPI assay, parameters 5 and 6 are related directly to the extraction process (Table S2a, Figure 11b). Parameter 8 covers other simple sample preparation steps, and the value of parameter Va results from the need to use additional equipment. Parameters 11 and IVb reflect the differences in flammability of the reagents used. Parameter II refers to the speed of determination and temperature conditions. Parameter III refers to the number of green chemistry principles met.

According to the AGREE test, the semi-automatic titration method with a camera (used in this work) obtained a slightly higher “greenness” rating than the potentiometric titration method, as well as the reference AAS method (Table S2a,b, Figure 11a). The advantage of semi-automatic camera titration over potentiometric titration is due to lower analyte consumption (parameter 2) and to measurement automation (parameter 5). The ComplexMoGAPI test, in turn, indicates increased operator safety in semi-automatic camera titration (parameters 13 and Vc in Figure 11b and Table S2a,b).

Compared to AAS, the advantages of the semi-authomatic titration method according to AGREE (Figure 11a, Table S2b) are a smaller amount of analyte collected for testing (criterion 2), fewer operations involved in the determination (criterion 4), lower energy consumption (criterion 9), lower consumption of toxic reagents (criterion 11), and greater operator safety (criterion 12). In turn, the advantage of the AAS method is greater measurement efficiency (criterion 8). The titrimetric method performs better when assessed with the ComplexMoGAPI test (Table S2b, Figure 11b), too. Titration is a direct method, while AAS requires ashing the sample (criterion 5). This implies an increased number of operations (criterion 8) and a greater energy input (criteria 12, II, and Vb). The ComplexMoGAPI test shows the advantage of titration over the AAS method, also in terms of reagent toxicity (criteria 11 and IVb), emission of harmful substances into the atmosphere (criteria 13 and Vc), and the degree of complexity of the measuring equipment (criterion Va).

### 2.5. Determination of Calcium in Dairy Products

Table 4 shows the exemplary results of calcium determination in cow milk samples from a local farm. Since cows are fed the same feed, observed differences in calcium content may be due to differences in breed, age, health, lactation stage, etc. The influence of these factors has been reported in the literature [14,28]. It is known that the milk of cows of different breeds differs in the content of casein and phospholipids [28]. As a result, larger amounts of these components result in higher concentrations of certain minerals, including calcium, which is bound to casein. According to [28], the exemplary calcium contents in cow milk are as follows: Jersey 146 mg/100 mL, Holstein 121 mg/100 mL, and Ayrshire 111 mg/100 mL. Taking into account that the variability of calcium content in milk of cows of the same breed is also large [28], the calcium content values determined (Table 4) can be considered typical.

Table 5 shows the calcium content in the commercial milk samples. For cow milk, the obtained values are in the typical range of 111 to 146 mg/100 mL [28]. There is a noticeable dependence of calcium content in cow’s milk on fat content. A comparison of samples LA-1 to LA-9 from the same producer shows that with increasing fat content, the amount of calcium in milk decreases. This trend is also observed in condensed milk (sample LA-1), which has a high fat content. Some differences in calcium content were observed in milk samples from different batches of the same manufacturer (the samples from LA-2 to LA-4 and from LA-5 to LA-7). It is characteristic that producers generally declare the same calcium content in milk, i.e., 120 mg/100 mL. This is probably an approximate and average value that does not account for changes in calcium content over time.

Literature reports on calcium content in goat milk are also variable, with average values reported being slightly higher than in cow milk, e.g., 134 mg/100 mL [29] or 130 mg/100 mL [30]. In raw goat milk, calcium content is 113 mg/100 mL [31] or 117 ± 6.7 mg/100 g [9], while it is 132 or 155 mg/100 mL in UHT milk [31]. Our results on calcium content in goat milk samples (Table 5) fall within the lower limit of this range.

Sheep milk was found to be rich in calcium (Table 5). The values from the literature are 198 mg/100 g [32] or 182 mg/100 mL [30]. This may be because sheep milk contains increased amounts of protein and phosphorus [32]. On the other hand, lower values of calcium content (110 ±5.7 mg/100 g) have also been reported [9]. Thus, the calcium content detected in the sheep milk sample (Table 5) seems to be typical.

**Figure 12:**
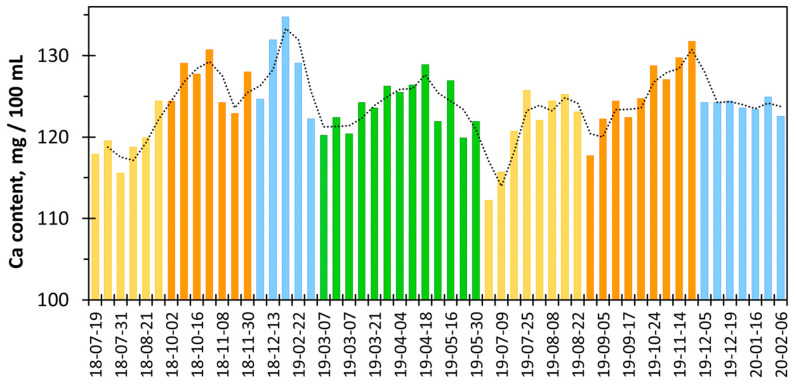
Calcium content in milk from the local farm [35] obtained using the calcein indicator vs. sample acquisition time (summer—yellow bars, autumn—orange bars, winter—blue bars, and spring—green bars).

Figure 12 shows seasonal variations in calcium content in cow milk samples from the same farm recorded over 1.5 years. The minimum values were recorded in July, while the maximum values were recorded in October to December. Literature reports confirm that the protein and calcium content in milk is lower in summer and higher in winter [28,33]. This is probably influenced by the way the cows are fed at different times of the year. At higher temperatures in summer, feed consumption decreases and water demand increases [33]. As a consequence, the calcium content in body fluids and milk decreases. In autumn and winter, feed is consumed in larger amounts and is also enriched with grains, cereals, and various plant ingredients [33]. The use of sodium supplements in feed may also cause a slight increase in calcium and magnesium concentrations in milk [34].

Figure 12 explains why the differences between the calcium content values measured and declared by the manufacturer can sometimes be quite significant. It seems that despite seasonal changes in calcium concentration, producers declare a constant, average amount of calcium (Table 5). A similar observation was made when examining the calcium content in mineral waters [22].

Table 6 shows calcium content in commercial dairy products. In most cases, the products contain calcium in typical amounts. For example, calcium content in yogurt was reported to be 110 mg/100 g [11]. The mean value of nine commercial yogurt samples was reported to be 115 ±12.8 mg/100 g [10]. However, some products contain higher amounts of calcium (e.g., samples KR-1, BN in Table 6). These samples are calcium-fortified products intended for people with special nutritional needs.

Calcium content in cream samples varies from 71.4 to 119.9 mg/100 g (Table 6). For comparison, the calcium content in cream has been reported to be 67.6 ±8.5 mg/100 g [14]. Typical content of calcium in cottage cheese is 100–125 mg Ca/100 g [2]. Similar to milk (Table 5), cream and cottage cheese (Table 6) reveal a negative correlation between calcium and fat contents. Samples richer in fat are generally poorer in calcium. This is because calcium is found mainly in the aqueous part of dairy products. The calcium content in powdered products is particularly high (Table 7).

When dissolved in the typical proportion of 30–35 g of powder per 250 mL, the calcium content in a drink obtained from milk powder becomes typical of cow milk. In turn, the concentration of calcium in the whey solution, i.e., a by-product of milk processing, will be low, similar to the literature value of 34.9 ± 1.2 mg/100 g [14].

Example titration curves for commercial products are provided in the Supplementary Materials, Figure S4a–g.

## 3. Materials and Methods

### 3.1. Reagents and Instrumentation

**Figure 13:**
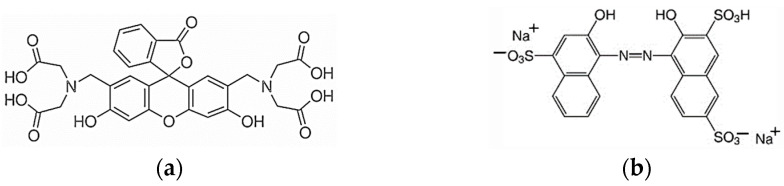
Structural formulas of indicators used: (**a**) calcein, (**b**) hydroxy naphthol blue.

The standard 0.025 M EDTA solution, the standard 0.01 M CaCl_2_ solution, and NaOH came from Chempur (Piekary Śląskie, Poland). The indicators were calcein and hydroxy naphthol blue (HNB) from Sigma-Aldrich (Saint Louis, MO, USA). Figure 13 shows the molecular structures of these indicators. To perform the measurements with the reference AAS method, the following reagents were used: 25% HNO_3_ suprapur (Merck, Darmstadt, Germany), a standard solution of Ca(NO_3_)_2_ in 0.5 M HNO_3_ of Ca concentration 1 g/L (Merck, Darmstadt, Germany), and LaCl_3_ (Sigma-Aldrich, Saint Louis, MO, USA). Dairy products were purchased in Polish stores.

### 3.2. Titration Procedure and Image Analysis

The measurement set for semi-automatic titration consisted of a Titronic 300 burette (SI Analytics, Burlington, MA, USA), a Logitech C270 webcam, and a computer [22]. The custom software ChemiON v. 1.4.8.5 was written in Java by Jan Lamkiewicz [36]. Titrations in the presence of the calcein indicator were performed in fluorescence mode, using the black background and an LED spotlight (360 lm, 6000 K) placed above the titration beaker. Titrations in the presence of the HNB indicator were performed in diffuse reflection mode, using the white background and an LED lamp (1260 lm, 4500 K) placed above the titration beaker.

Test solutions with defined calcium content were prepared by diluting exact volumes of standard 0.01 M CaCl_2_ solution with deionized water or 1% aqueous milk solution. Dispersions of liquid or semi-liquid dairy products were obtained by diluting an exact volume/mass of product with deionized water (1 mL of milk or 1 g of other products per 100 mL). The cottage cheese was dispersed in 3% NaOH. Before the titration procedure, the pH of the sample was adjusted to 12.5 with 8 M NaOH solution, and the exact volume of the indicator was added.

In the titration procedure, standard EDTA solution was added to 100 mL of the examined solution in increments of 0.02 or 0.05 mL. The camera recorded the images of the reaction beaker 10 s after each dose step. The images of the reaction mixture were analyzed with the software ChemiON. The sampling area was set by the operator. Each of the RGB color components was averaged over the sampling area. Transformation of the RGB values (8-bit integer) to the Hue parameter (degrees) was carried out according to the HSI color model. The variance of the endpoint volume was calculated from the results of 8–10 determinations.

### 3.3. AAS Measurements

About 5 g of accurately weighed (±0.0001 g) dairy product was mineralized in an oven. The oven temperature was increased gradually at a rate of 50 °C/h to 550 °C, and then the sample was kept at this temperature for 6 h. The ash obtained was dissolved in 2 mL of HNO_3_ solution and left for 2 h. Then, the sample was diluted with deionized water to obtain a calcium concentration between 1 and 20 mg/L (the range of the standard curve). Finally, 10% *v*/*v* LaCl_3_ solution of 20 g La/L was added to eliminate phosphate interference. The AAS spectral measurements were performed using an iCE 3000 Series AA (Thermo Scientific, Waltham, MA, USA) spectrophotometer at a wavelength of 422.7 nm.

## 4. Conclusions

This study aimed to simplify the complexometric titration procedure for the determination of calcium in milk and dairy products. Turbid dairy dispersions were used as a white background, making it easier to detect titration endpoints with a webcam. This made the protein coagulation step unnecessary, and sample preparation became simpler. A convenient and inexpensive measurement set was used. Two calcium-sensitive indicators were tested: calcein and hydroxy naphthol blue. Correct indicator dosage and appropriate sample dilution are required for precise recognition of the titration endpoint by the camera. When using the calcein indicator, the suitable color parameters are Hue, Green, and Blue. When using the hydroxy naphthol blue indicator, the suitable color parameters are Hue, Red, and Green. The result of image analysis practically does not depend on the size of the sampling area selected by the operator. The determined calcium contents are consistent with the results of the reference AAS method. RSD values for milk and chosen dairy products are in the range from 0.45 to 2.48%. Measured values of calcium content in milk and dairy products may differ from the values declared by manufacturers. Long-term measurement of calcium concentration in cow milk revealed seasonal trends.

The advantage of the developed complexometric titration method is its environmental friendliness, primarily due to the elimination of the sample preparation stage and the low energy consumption of the titration method. In this respect, the semi-automatic complexometric titration with a camera proved to be significantly better than other titration methods and the reference AAS method.

The described titration method is ready to be implemented on any MS Windows computer. In a commercial application, little operator training will be required to adjust lighting conditions. To use this method in fully automated mode with an autosampler, it is necessary to upgrade the software used. The possible limitations of the webcam detector are sensitivity to ambient lighting, operator bias in sampling area, and matrix-dependent variations. To minimize the effect of ambient light conditions, intense artificial lighting can be used. To eliminate any operator bias when selecting the color sampling area, a well-illustrated manual will be useful. The matrix effect may be completely detrimental in the case of colored dairy products, such as fruit yogurts. In this case, an additional coagulation step will be necessary.

## Figures and Tables

**Table 1:** Advantages and disadvantages of the most commonly used techniques for Ca determination in milk and dairy products.

Technique	Advantages	Disadvantages
AAS	○ Possibility of determining multiple elements ○ Less expensive than ICP ○ Simple to operate	○ Time-consuming (sample mineralization) ○ Cost of La as a releasing agent ○ One analyte at a time
ICP-OES	○ Simultaneous measurement of multiple elements ○ Fast analysis ○ High accuracy	○ Time-consuming (sample mineralization) ○ Sensitive to matrix components
INNA	○ No sample preparation ○ Simultaneous measurement of multiple elements ○ Sensitive technique	○ High costs of equipment ○ Labor-intensive
ISE	○ No sample preparation ○ Ease of use ○ Low cost ○ Short response time	○ Calibration solutions required ○ One analyte at a time ○ Matrix effects
LIBS	○ No sample preparation ○ Fast analysis ○ Simultaneous measurement of multiple elements ○ Real-time technique	○ The influence of the matrix on the measurement result ○ Limited use for liquid samples
XRF	○ No sample preparation ○ Simultaneous measurement of multiple elements ○ Fast analysis ○ Reagent-free technique ○ Simple to operate	○ Lower sensitivity compared to ICP ○ High costs of equipment
Complexometric titration	○ Low costs of equipment ○ Low energy consumption ○ Simple to operate ○ No sample preparation when using an ISE detector	○ Large consumption of the analyte ○ One analyte at a time ○ Labor-intensive when using the protein elimination procedure ○ Problem with the visual recognition of the endpoint

**Table 2:** Statistical parameters of calcium determination in water using hydroxyl naphthol blue at 4 mmol/L.

**Ca Amount, μmol**	**Signal**	**SD, μmol**	**RSD, %**	**Recovery, %**
50	Red	0.80	1.50	107.5
50	Blue	0.50	0.95	105.5
50	Hue	0.00	0.00	105.0
5	Red	0.34	3.00	113.0
5	Blue	0.32	5.85	108.5
5	Hue	0.16	3.01	106.5

**Table 3:** (a). RSD of calcium determination in chosen products using calcein of 8 μmol/L and hydroxyl naphthol blue of 8 mmol/L. Volume of milk was 1 mL, and mass of other products was approximately 1 g. (b). Two-sided *p*-values obtained from the *t*-test for calcium determination results presented in Table 3a. Confidence interval is 95%, significance threshold is 0.05.

**(a)**											
**Product**	**Titration**								**AAS**		
	**Signal**	**Calcein**			**HNB**						
		Ca Content *	**RSD, %**		Ca Content *		**RSD, %**		Ca Content *		**RSD, %**
milk (fat cont. 2%)	Red	-	-		129.38		1.47		120.8		0.6
	Green	125.88	1.32		134.64		1.78				
	Blue	138.85	2.96		-		-				
	Hue	128.13	1.32		128.13		1.63				
cream (fat cont. 30%)	Red	-	-		76.45		2.48		76.1		0.93
	Green	71.36	1.43		89.90		1.82				
	Blue	73.69	1.10		-		-				
	Hue	71.37	2.22		77.62		1.63				
yogurt (fat cont. 7.5%)	Red	-	-		126.17		0.45		126.0		0.88
	Green	126.66	0.56		131.33		0.33				
	Blue	132.62	0.68		-		-				
	Hue	124.81	1.14		137.04		0.95				
**(b)**											
**Product**	**Signal**	**Calcein/Green**		**Calcein/Hue**		**HNB/Red**		**HNB/Hue**		**AAS**	
milk	calcein/Green	-		0.0246		0.0025		0.0422		<0.0001	
	calcein/Hue	0.0246		-		0.2145		1.0000		<0.0001	
	HNB/Red	0.0025		0.2145		-		0.2609		<0.0001	
	HNB/Hue	0.0422		1.0000		0.2609		-		<0.0001	
	AAS	<0.0001		<0.0001		<0.0001		<0.0001		-	
cream	calcein/Green	-		0.9863		0.0004		<0.0001		<0.0001	
	calcein/Hue	0.9863		-		0.0010		<0.0001		<0.0001	
	HNB/Red	0.0004		0.0010		-		0.1890		0.6547	
	HNB/Hue	<0.0001		<0.0001		0.1890		-		0.0136	
	AAS	<0.0001		<0.0001		0.6547		0.0136		-	
yogurt	calcein/Green	-		0.0290		0.2889		<0.0001		0.2959	
	calcein/Hue	0.0290		-		0.0743		<0.0001		0.1728	
	HNB/Red	0.2889		0.0743		-		<0.0001		0.7480	
	HNB/Hue	<0.0001		<0.0001		<0.0001		-		<0.0001	
	AAS	0.2959		0.1728		0.7480		<0.0001		-	

* mg/100 mL for milk, mg/100 g for other products.

**Table 4:** Determined calcium content in raw milk of different cow breeds. Indicator: HNB.

Cattle Breed	Fat Content, %	Ca Content, mg/100 mL
Jersey *	≥5.4	136
Simmental *	4.2	126
Holstein–Friesian red-white *	4.3	102
Holstein–Friesian black-white *	4.1	123
Holstein–Friesian black-white	4.2	121

* The cows were from the same farm.

**Table 5:** Determined calcium content in commercial milk samples. Indicator: calcein or HNB.

Origin	Brand	Fat Content, %	Ca Content Declared, mg/100 mL	Ca Content Determined, mg/100 mL
cow	EK	3.9	-	147
	AU	3.2	105	117
	KC-1	3.2	-	115
	MD-1	3.2	120	136
	MI	3.2	-	125
	MU	3.2	-	108
	WY	3.2	105	130
	LA-1	7.5	-	271
	LA-2	3.2	120	102
	LA-3	3.2	120	115
	LA-4	3.2	120	116
	LA-5	2.0	120	120
	LA-6	2.0	120	124
	LA-7	2.0	120	126
	LA-8	0.5	120	126
	LA-9	0.0	120	130
	ML-1 *	1.5	105	101
	LO-1	3.2	120	108
	RO	3.2	-	118
	UC	3.2	-	104
	WS	3.2	121	116
goat	DA	2.5	-	118
	DA *	2.5	-	120
sheep	TE	1.7	185	181

* Without lactose.

**Table 6:** Determined calcium content in dairy products. Indicator: calcein or HNB.

Category	Brand	Fat Content, %	Ca Content Declared, mg/100 g	Ca Content Determined, mg/100 g
Yogurt	BG	7.5	-	126.7
	PZ-1	3.1	120.0	144.7
	FR	3.0	120.0	151.5
	FI	2.0	-	108.2
	KR-1	2.0	240	281
	BN	1.5	150.0	150.3
	LO-2	1.5	-	108.2
	RO	1.5	-	116.2
	PZ-2	1.4	120	115.2
Kefir	BN	3.0	120	128.3
	MD-2	1.5	145	118.7
Buttermilk	KR-2	4.0	-	140.3
	MR	1.5	130.0	117.2
Cream	PZ-3	30	-	84.2
	PZ-4	30	-	71.4
	PZ-5	30	-	72.1
	PZ-6	18	-	84.2
	PZ-7	12	-	87.2
	PZ-8	12	-	80.2
	MD	10	-	105.3
	KA	10	-	119.9
Cottage cheese	DB	5.0	-	99.2
	OM	5.0	-	115.2
	KC-2	4.2	-	110.2
	KK	4.0	-	108.2
	LO-3	3.5	-	106.22

**Table 7:** Determined calcium content in powdered products. Indicator: calcein.

Category	Brand	Fat Content, %	Ca Content Declared, mg/100 g	Ca Content Determined, mg/100 g
Milk	ML-2	26.0	-	941
Buttermilk	ML-3	7.0	1050	1074
Whey	ML-4	2.1	460	608
Demineralized whey	ML-5	1.0	-	361

## Data Availability

All data are included in the article and Supplementary Materials.

## Supplementary Materials

The following supporting information can be downloaded at: https://www.mdpi.com/article/10.3390/molecules30173553/s1, Figure S1. (a) Graphs of the Blue values vs. EDTA volume obtained in water solution containing 1 %v/v of milk with addition of calcium ion, using the calcein indicator (8 μmol/L). (b) The dependences of titrant volume on calcium amount derived for Blue signal in milk water solutions of calcium and water solutions of calcium. Figure S2. (a) Graphs of the Blue values vs. EDTA volume obtained using the hydroxyl naphtol blue indicator (4 mmol/L). (b) Corresponding dependences of titrant volume on calcium amount. Figure S3. (a) Graphs of the Green values vs. EDTA volume obtained in water solution containing 1 %v/v of milk with addition of calcium ion, using the hydroxyl naphtol blue indicator (8 mmol/L ). (b) Corresponding dependences of titrant volume on calcium amount. Figure S4(a)–(g). Exemplary titration graphs. Table S1. Results of analytic methods comparison applied for liquid or semi-liquid dairy products using the AGREE test. Table S2a. Results of analytic methods comparison applied for liquid or semi-liquid dairy products using the ComplexMoGAPI test - titration with extraction and potentiometric titration. Table S2b. Results of analytic methods comparison applied for liquid or semi-liquid dairy products using the ComplexMoGAPI test – AAS and semi-automatic titration with cam.

## Abbreviations

The following abbreviations are used in this manuscript:

EDTA	Ethylenediaminetetraacetic Acid
HNB	Hydroxy Naphthol Blue
RGB	Red–Green–Blue
