# Supplementary material for: Application of the Semi-Automatic Titration Method Using a Webcam for the Determination of Calcium in Milk and Dairy Products
Source: Molecules. 2025 Aug 30;30(17):3553. doi: 10.3390/molecules30173553 (PMC12430243; doi:10.3390/molecules30173553)
Supplement: Supplementary file 1 [file molecules-30-03553-s001.zip › molecules-3802908-supplementary.pdf]

molecules

MDPI

Supplementary materials

Application of the Semi-Automatic Titration Method Using a Webcam for the Determination of Calcium in Milk and Dairy Products

Alexander Shyichuk 1,2, Dorota Ziółkowska 1,* , Iryna Shyychuk 1 and Maria Kowalska 1

1 Faculty of Chemical Technology and Engineering, Bydgoszcz University of Science and Technology, Seminaryjna 3, 85-326 Bydgoszcz, Poland; szyjczuk@pbs.edu.pl (A.S.); iryna.shyychuk@pbs.edu.pl (I.S.); maria.kowalska@pbs.edu.pl (M.K.)

2 Educational and Scientific Center of Materials Science and Nanotechnology, Vasyl Stefanyk Precarpathian National University, 76018 Ivano-Frankivsk, Ukraine

* Correspondence: dorota_z@pbs.edu.pl

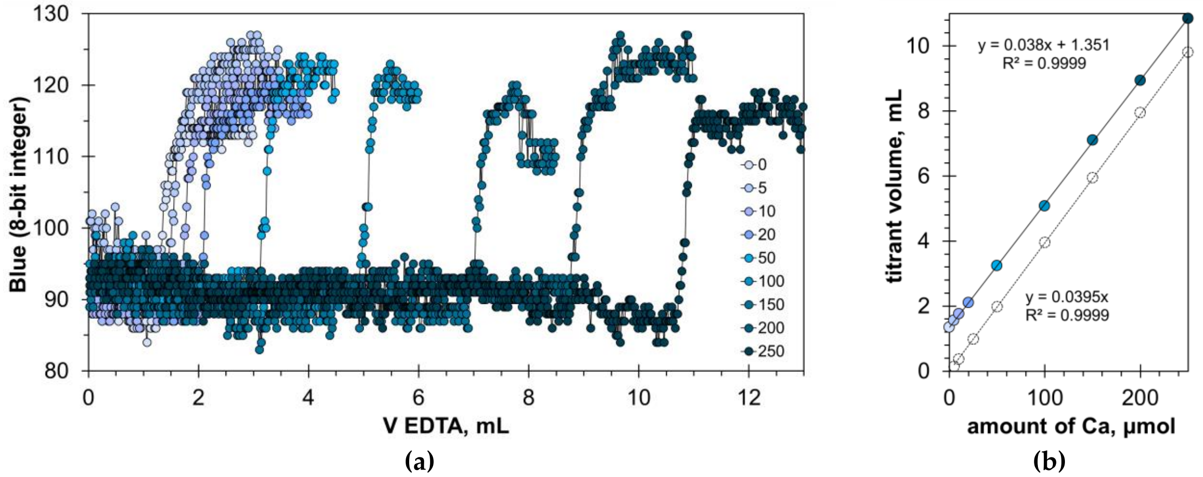

Figure S1. (a) Graphs of the Blue values vs. EDTA volume obtained in water solution containing 1 %v/v of milk with addition of calcium ion, using the calcein indicator (8 μmol/L). The amounts of calcium ions are indicated in μmol. **(b)** The dependences of titrant volume on calcium amount derived for Blue signal in milk water solutions of calcium (blue points) and water solutions of calcium (colorless points).

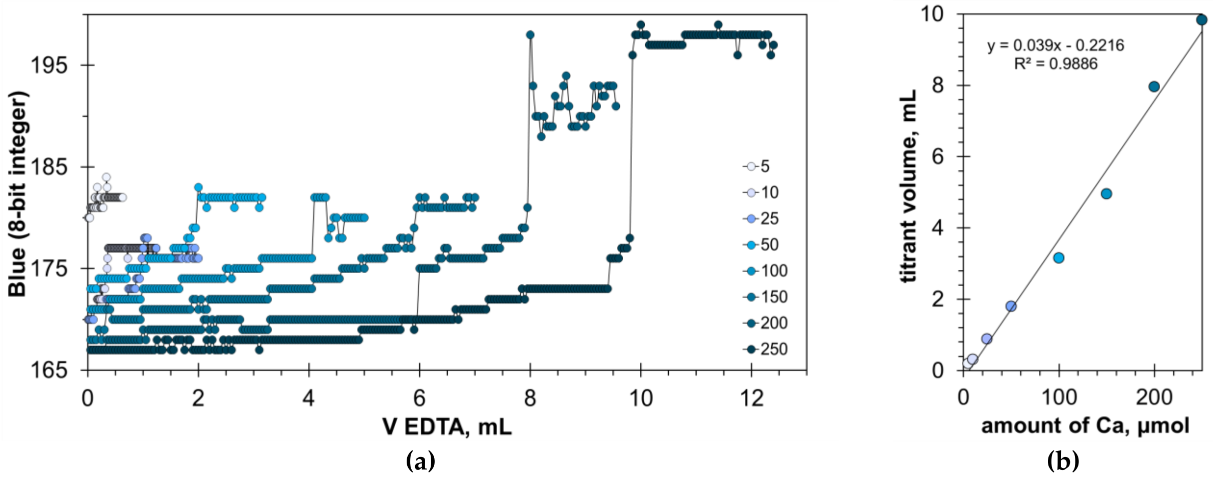

Figure S2. (a) Graphs of the Blue values vs. EDTA volume obtained using the hydroxyl naphtol blue indicator (4 mmol/L). The amounts of calcium ions are indicated in μmol. **(b)** Corresponding dependences of titrant volume on calcium amount.

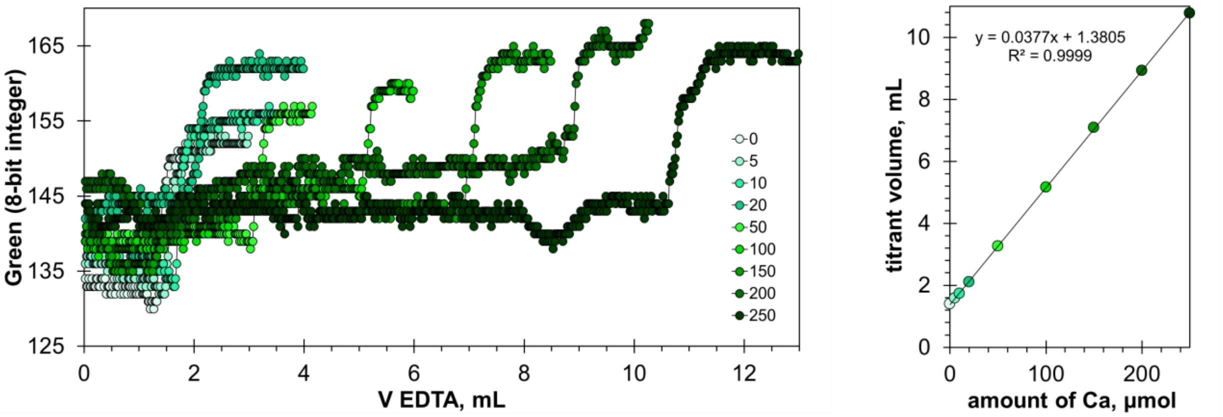

(a) **(b)**

Figure S3. **(a)** Graphs of the Green values vs. EDTA volume obtained in water solution containing 1 %v/v of milk with addition of calcium ion, using the hydroxyl naphtol blue indicator (8 mmol/L). The amounts of calcium ions are indicated in μmol. **(b)** Corresponding dependences of titrant volume on calcium amount.

Table S1. Results of analytic methods comparison applied for liquid or semi-liquid dairy products using the AGREE test. The parameter weight is given in brackets. Source of assay: AAS - this paper, titration with extraction - ISO-12081-2010*), potentiometric titration - Metrohm Application Bulletin 235/3 e**), semi-automatic titration with cam - this paper.

No	Principle	AAS	Titration with extraction	Potentiometric titration	Semi-automatic titration with cam.
1	Direct Analytical Techniques Should Be Applied to Avoid Sample Treatment	Off-line analysis (2)	Off-line analysis (2)	Off-line analysis (2)	Off-line analysis (2)
2	Minimal Sample Size and Minimal Number of Samples Are Goals	5 mL (1)	20 mL (1)	10 mL (1)	1 mL (1)
3	In Situ Measurements Should Be Performed	Off-line (2)	Off-line (2)	Off-line (2)	Off-line (2)
4	Integration of Analytical Processes and Operations Saves Energy and Reduces the Use of Reagents	6 steps (2)	13 steps (2)	4 steps (2)	4 steps (2)
5	Automated and Miniaturized Methods Should Be Selected	Semi-automatic, not miniaturized (2)	Manual, non-miniaturized (2)	Manual, non-miniaturized (2)	Semi-automatic, not miniaturized (2)
6	Derivatization Should Be Avoided	No derivatization (2)	Derivatization (2)	No derivatization (2)	No derivatization (2)
7	Generation of a Large Volume of Analytical Waste Should Be Avoided and Proper Management of Analytical Waste Should Be Provided	100 mL (2)	80 mL	61 mL (2)	100 mL (2)
8	Multianalyte or Multiparameter Methods Are Preferred versus Methods Using One Analyte at a Time	1 analyte / run, 60 samples / h (2)	1 analyte / run, 0.2 sample / h (2)	1 analyte / run, 4 sample / h (2)	1 analyte / run, 4 sample / h (2)
9	The Use of Energy Should Be Minimized	Flame atomic absorption spectrometry (2)	Titration (2)	Titration (2)	Titration (2)

10	Reagents Obtained from Renewable Source Should Be Preferred	None of the reagents are from bio-based sources (2)	None of the reagents are from bio-based sources (2)	None of the reagents are from bio-based sources (2)	None of the reagents are from bio-based sources (2)
11	Toxic Reagents Should Be Eliminated or Replaced	Toxic reagents: 0.60g (2)	Toxic reagents: 7.2 g (2)	Toxic reagents: 0.34 g (2)	Toxic reagents: 0.36 g (2)
12	The Safety of the Operator Should Be Increased	Toxic to aquatic life; highly flammable; explosive (2)	Toxic to aquatic life, corrosive (2)	Toxic to aquatic life, corrosive (2)	Toxic to aquatic life, corrosive (2)

***) [27]** https://cdn.standards.iteh.ai/samples/55776/904be797336643a7ab3273cba2fcc596/ISO-12081-2010.pdf (Accessed on 21.07.2025)

****) [19]** https://www.metrohm.com/content/dam/metrohm/shared/documents/application-bulletins/AB-235_3.pdf (Accessed on 21.07.2025)

Table S2a. Results of analytic methods comparison applied for liquid or semi-liquid dairy products using the ComplexMoGAPI test. The source of the methods is given in Table S1.

No	Criterion	Score	
		**Titration with extraction**	**Potentiometric titration**
		SAMPLE PREPARATION	
1	Collection	Off-line	Off-line
2	Preservation	None	None
3	Transport	Required	Required
4	Storage	Under normal conditions	Under normal conditions
5	Type of method: direct or indirect	Extraction required	No sample preparation
6	Scale of extraction	Macro-extraction	Not applicable
7	Solvents/reagents used	Non-green solvents/reagents used	Non-green solvents/reagents used
8	Additional treatments	Simple treatments	None
		REAGENTS AND SOLVENTS	
9	Amount	10-100 (10-100 g)	10-100 mL (10-100 g)
10	Health hazard	Moderately toxic; could cause temporary incapacitation; NFPA = 2 or 3	Moderately toxic; could cause temporary incapacitation; NFPA = 2 or 3
11	Safety hazard	Highest NFPA flammability, instability score of 2 or 3	Highest NFPA flammability, instability score of 0 or 1.
		INSTRUMENTATION	
12	Energy	≤0.1 kW h per sample	≤0.1 kW h per sample
13	Occupational hazard	Emission of vapours to the atmosphere	Emission of vapours to the atmosphere
14	Waste	>10 mL (>10 g)	>10 mL (>10 g)
15	Waste treatment	No treatment	No treatment
16	QUANTIFICA-TION	Procedure for qualification and quantification	Procedure for qualification and quantification
		YIELD AND CONDITIONS	
I	Yield	>89%	>89%
II	Temperature/time	Heating, >1 h	Room temperature, <1 h
		RELATION TO GREEN ECONOMY	

III	Number of rules met	1	3
		REAGENTS AND SOLVENTS	
IVa	Health hazard	Moderately toxic; could cause temporary incapacitation; NFPA = 2 or 3	Moderately toxic; could cause temporary incapacitation; NFPA = 2 or 3
IVb	Safety hazard	Highest NFPA flammability, instability score of 2 or 3	Highest NFPA flammability, instability score of 0 or 1
		INSTRUMENTATION	
Va	Technical setup	Additional setups / semi-advanced instruments used	Common setup
Vb	Energy	≤0.1 kW h per sample	≤0.1 kW h per sample
Vc	Occupational hazard	Emission of vapours to the atmosphere	Emission of vapours to the atmosphere
		WORKUP AND PURIFICATION	
VIa	Workup and purification of the end product	Not applicable	Not applicable
VIb	Purity	Not applicable	Not applicable
	E-FACTOR	4	6

Table S2b. Results of analytic methods comparison applied for liquid or semi-liquid dairy products using the ComplexMoGAPI test. The source of the methods is given in Table S1.

No	Criterion	Score	
		**AAS**	**Semi-automatic titration with cam**
		SAMPLE PREPARATION	
1	Collection	Off-line	Off-line
2	Preservation	None	None
3	Transport	Required	Required
4	Storage	Under normal conditions	Under normal conditions
5	Type of method: direct or indirect	Simple procedures	No sample preparation
6	Scale of extraction	Not applicable	Not applicable
7	Solvents/reagents used	Non-green solvents/reagents used	Non-green solvents/reagents used
8	Additional treatments	Advanced treatments (mineralization)	None
		REAGENTS AND SOLVENTS	
9	Amount	10-100 (10-100 g)	10-100 mL (10-100 g)
10	Health hazard	Moderately toxic; could cause temporary incapacitation; NFPA = 2 or 3	Moderately toxic; could cause temporary incapacitation; NFPA = 2 or 3
11	Safety hazard	Highest NFPA flammability, instability score is 4	Highest NFPA flammability, instability score of 0 or 1
		INSTRUMENTATION	
12	Energy	>1.5 kW h per sample	≤0.1 kW h per sample
13	Occupational hazard	Emission of vapours to the atmosphere	Hermetic sealing of analytical process
14	Waste	>10 mL (>10 g)	>10 mL (>10 g)

15	Waste treatment	No treatment	No treatment
16	QUANTIFICA-TION	Procedure for qualification and quantification	Procedure for qualification and quantification
		YIELD AND CONDITIONS	
I	Yield	>89%	>89%
II	Temperature/time	Heating, >1h	Room temperature, <1 h
		RELATION TO GREEN ECONOMY	
III	Number of rules met	1	3
		REAGENTS AND SOLVENTS	
IVa	Health hazard	Moderately toxic; could cause temporary incapacitation; NFPA = 2 or 3	Moderately toxic; could cause temporary incapacitation; NFPA = 2 or 3
IVb	Safety hazard	Highest NFPA flammability, instability score is 4	Highest NFPA flammability, instability score of 0 or 1
		INSTRUMENTATION	
Va	Technical setup	Additional setups / semi-advanced instruments used	Common setup
Vb	Energy	>1.5 kW h per sample	≤0.1 kW h per sample
Vc	Occupational hazard	Emission of vapours to the atmosphere	Hermetic sealing of analytical process
		WORKUP AND PURIFICATION	
VIa	Workup and purification of the end product	Not applicable	Not applicable
VIb	Purity	Not applicable	Not applicable
	E-FACTOR	24	4

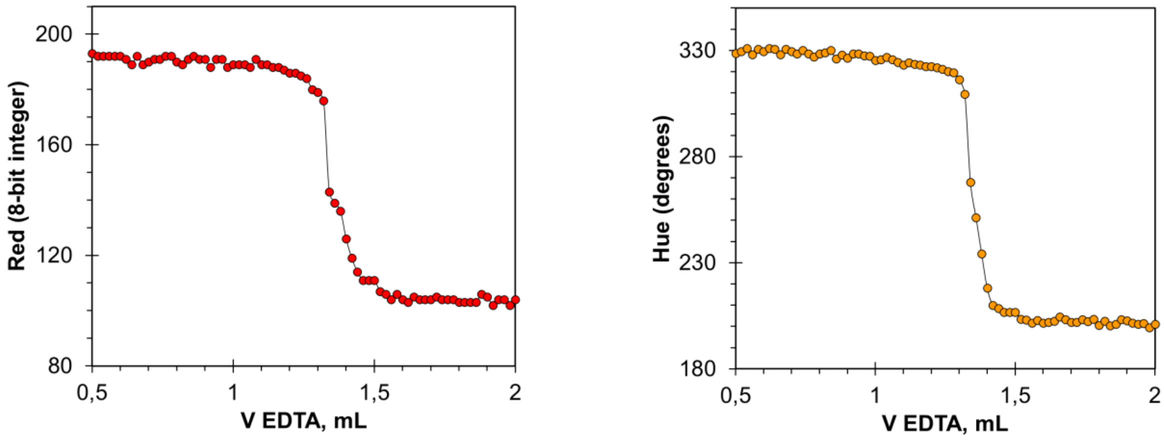

Figure S4(a). Titration graphs of caw milk (fat cont. 2%). Indicator: HNB.

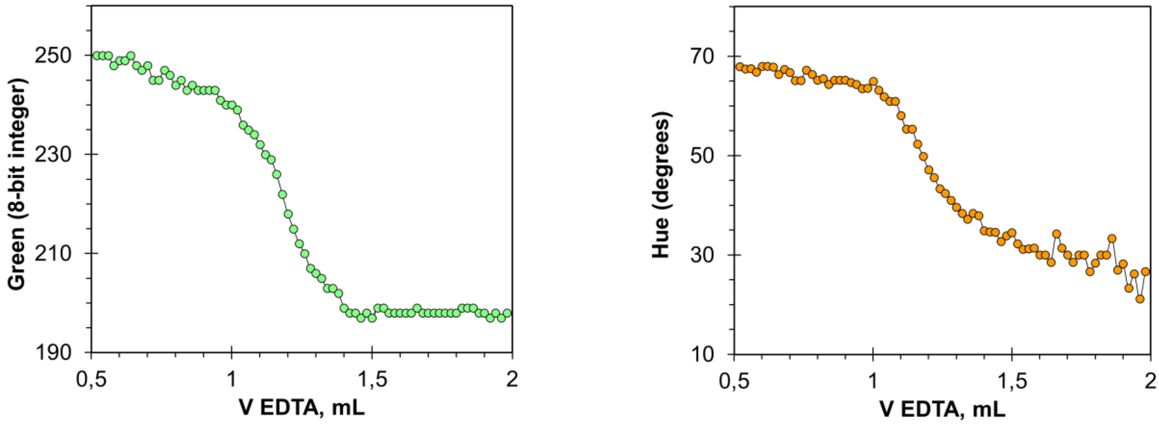

Figure S4(b). Titration graphs of goat milk (brand DA). Indicator: calcein.

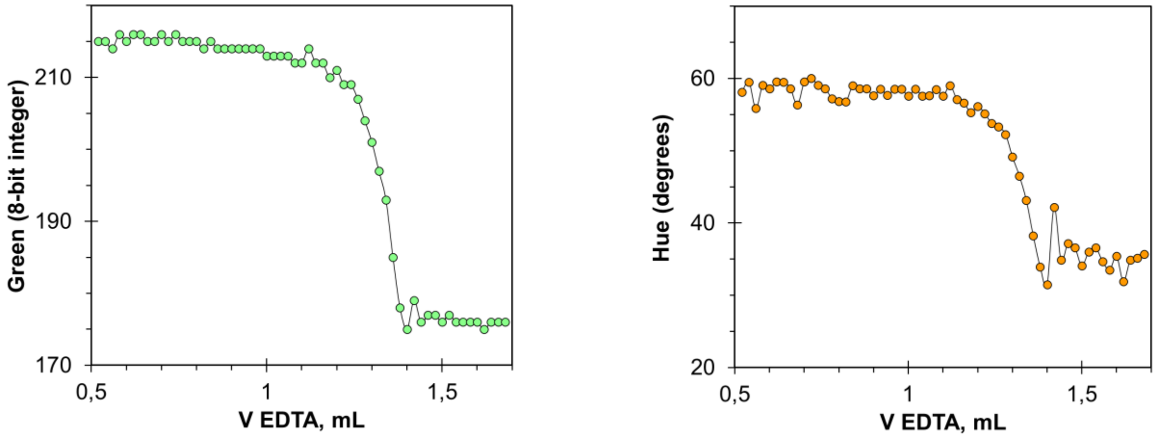

Figure S4(c). Titration graphs of powdered milk (brand ML-2). Sample mass 0.14 g. Indicator: calcein.

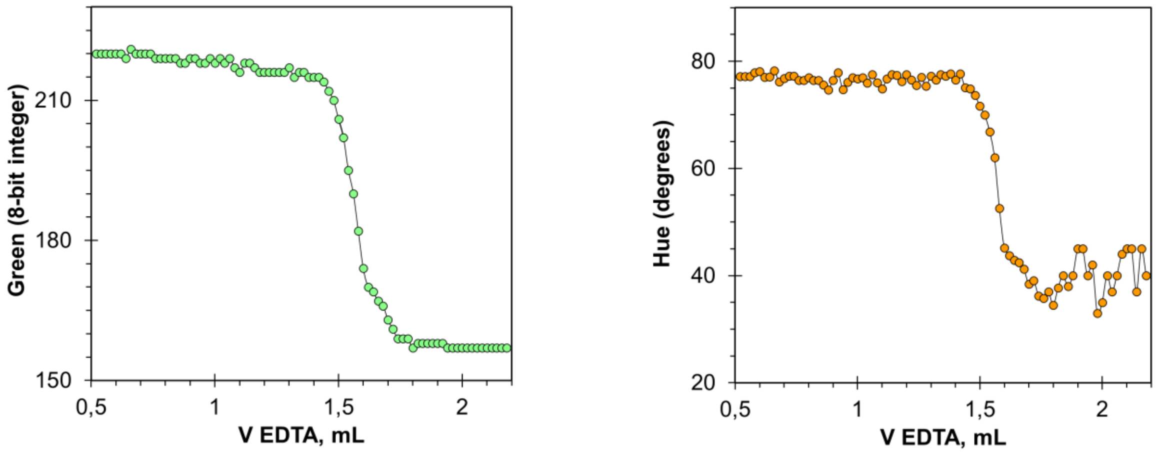

Figure S4(d). Titration graphs of yoghurt (brand PZ-1). Indicator: calcein.

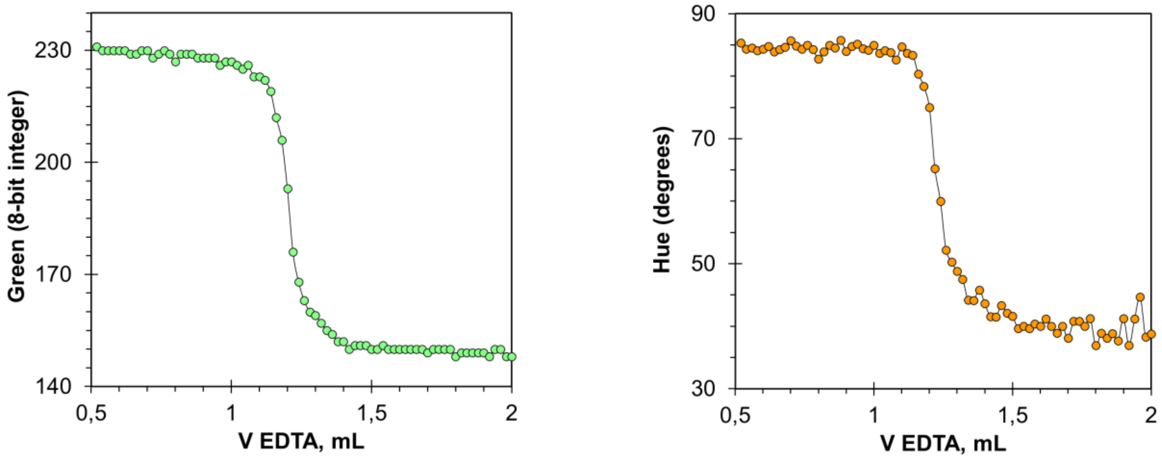

Figure S4(e). Titration graphs of kefir (brand MD-2). Indicator: calcein.

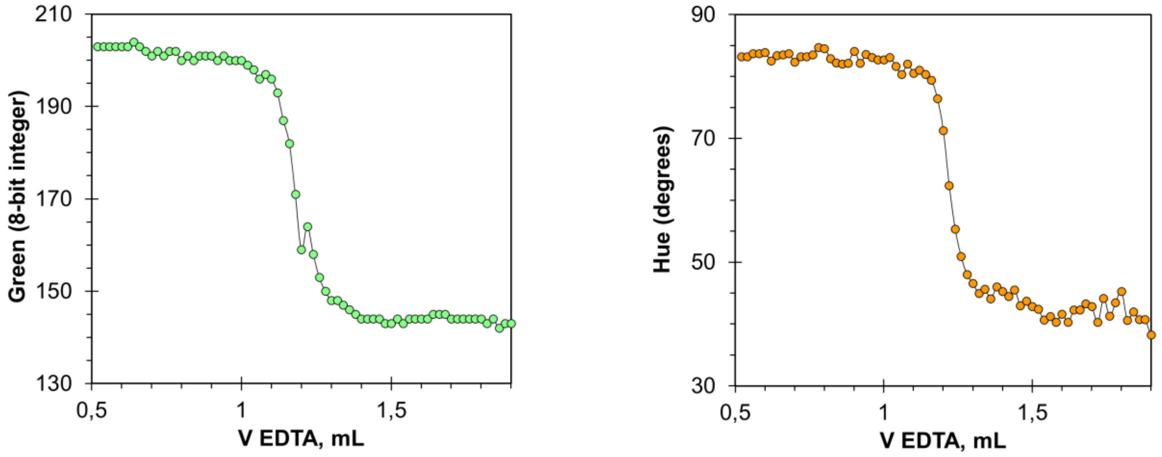

Figure S4(f). Titration graphs of buttermilk (brand MR). Indicator: calcein.

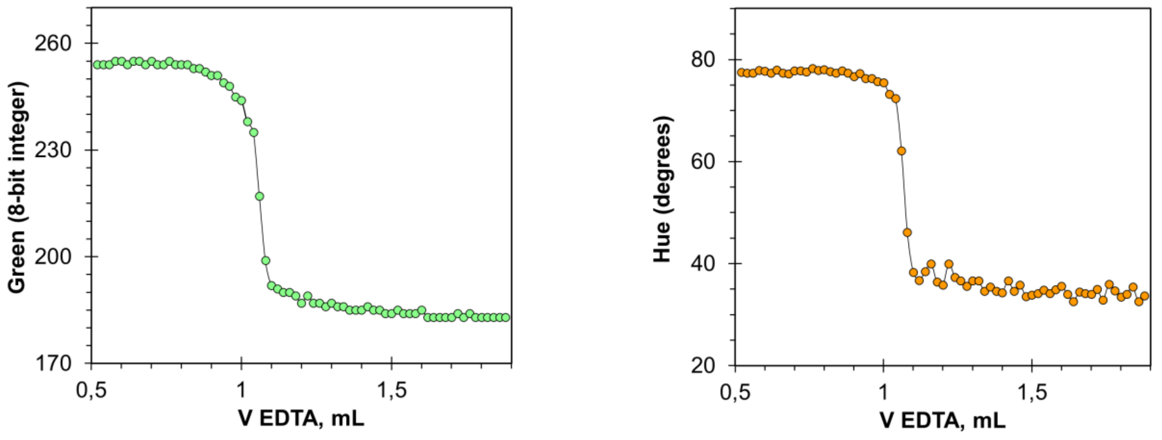

Figure S4(g). Titration graphs of cream (brand MD). Indicator: calcein.
